# Self‐Propelled Multifunctional Microrobots Harboring Chiral Supramolecular Selectors for “Enantiorecognition‐on‐the‐Fly”

**DOI:** 10.1002/anie.202116090

**Published:** 2022-02-09

**Authors:** Jose Muñoz, Mario Urso, Martin Pumera

**Affiliations:** ^1^ Future Energy and Innovation Laboratory Central European Institute of Technology Brno University of Technology 61200 Brno Czech Republic; ^2^ Center for Advanced Functional Nanorobots Dept. of Inorganic Chemistry Faculty of Chemical Technology University of Chemistry and Technology 16628 Prague Czech Republic; ^3^ Department of Medical Research China Medical University Hospital China Medical University Taichung 40402 Taiwan

**Keywords:** Chiral Analysis, Cyclodextrin, Magnetic Micromotors, Nickel Microrockets, Quantum Dots

## Abstract

Herein, a general procedure for the synthesis of multifunctional MRs, which simultaneously exhibit i) chiral, ii) magnetic, and iii) fluorescent properties in combination with iv) self‐propulsion, is reported. Self‐propelled Ni@Pt superparamagnetic microrockets have been functionalized with fluorescent CdS quantum dots carrying a chiral host biomolecule as β‐cyclodextrin (β‐CD). The “on‐the‐fly” chiral recognition potential of MRs has been interrogated by taking advantage of the β‐CD affinity to supramolecularly accommodate different chiral biomolecules (i.e., amino acids). As a proof‐of‐concept, tryptophan enantiomers have been discriminated with a dual‐mode (optical and electrochemical) readout. This approach paves the way to devise intelligent cargo micromachines with “built‐in” chiral supramolecular recognition capabilities to elucidate the concept of “enantiorecognition‐on‐the‐fly”, which might be facilely customized by tailoring the supramolecular host–guest encapsulation.

## Graphical abstract

Multifunctional microrobots, made of Ni@Pt microrockets, CdS quantum dots, and a host β‐cyclodextrin selector, have been engineered that simultaneously exhibit i) self‐propulsion, ii) fluorescence, and iii) magnetic properties in combination with iv) supramolecular chiral recognition for “enantiorecognition‐on‐the‐fly” approaches. As a model chiral target, tryptophan enantiomers have been discriminated using a bimodal (optical and electrical) readout.

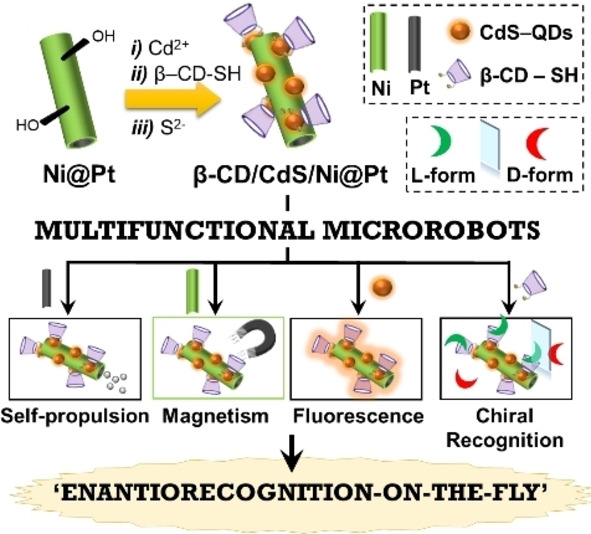

## Introduction

Self‐propelled microrobots (MRs) are at the forefront of materials science and nanotechnology owing to their capability to convert the surrounding energy into powerful motion, making it possible to perform a wide range of autonomous tasks in previously inaccessible locations while accelerating processes conventionally limited by diffusion.[^1^, ^2^, ^3^] In particular, the autonomous navigation of MRs represents one of the most exciting prospects in contemporary analytical nanotechnology for smart chemical bio‐sensing.[^4^, ^5^, ^6^] The motion‐based biosensing strategy allows “on‐the‐fly” molecular recognition through their continuous movement around the sample that induces efficient fluid mixing and greatly accelerates target–receptor interactions. For example, tuning “on‐the‐fly” molecular recognition MRs with a superparamagnetic core material is an appealing way to produce cargo micromachines since they can be easily separated/collected from complex matrices by simply utilizing an external magnet.^[7]^ This approach is especially useful in electroanalysis by employing magneto electrodes.[^8^, ^9^] Additionally, fluorescent semiconductor nanocrystals, also named as quantum dots (QDs), have a strong characteristic spectral emission and have recently attracted enormous interest in the field of MRs for optical detection strategies.[^10^, ^11^] However, the design and fabrication of smart self‐propelled MRs that simultaneously contain more than one functional component is a challenge,[^12^, ^13^] which might be of great potential for target determination with multimodal (e.g., electrical and optical) readout.

Chiral molecular structures are ubiquitous in nature, and their enantiomeric forms usually present different metabolic and pharmacological effects in biological systems.[^14^, ^15^] Therefore, the development of enantiorecognition agents is of essential importance in life sciences. One of the bases to achieve enantiorecognition platforms must be focused on engineering supramolecular recognition architectures containing active sites which exhibit different binding affinities for enantiomers.[^16^, ^17^] In this regard, chiral host molecules like cyclodextrins (CDs) have emerged as extremely versatile supramolecular cargo for enantioselective analyses because of their capability to form stable host–guest complexes in aqueous media with a wide range of small guest targets.^[18]^ To date, different CD‐based hybrid nanocomposites built on independently coating passive superparamagnetic nanoparticles or QDs have already demonstrated high potential for the enantiodiscrimination of chiral biomolecules as amino acids (e.g., tryptophan, Trp).[^19^, ^20^] Nonetheless, their incorporation on self‐propelled MRs as chiral host selectors is nowadays an unexplored field which may yield to the achievement of supramolecular cargo micromachines displaying unprecedented “enantiorecognition‐on‐the‐fly” capabilities.

**Scheme 1:**
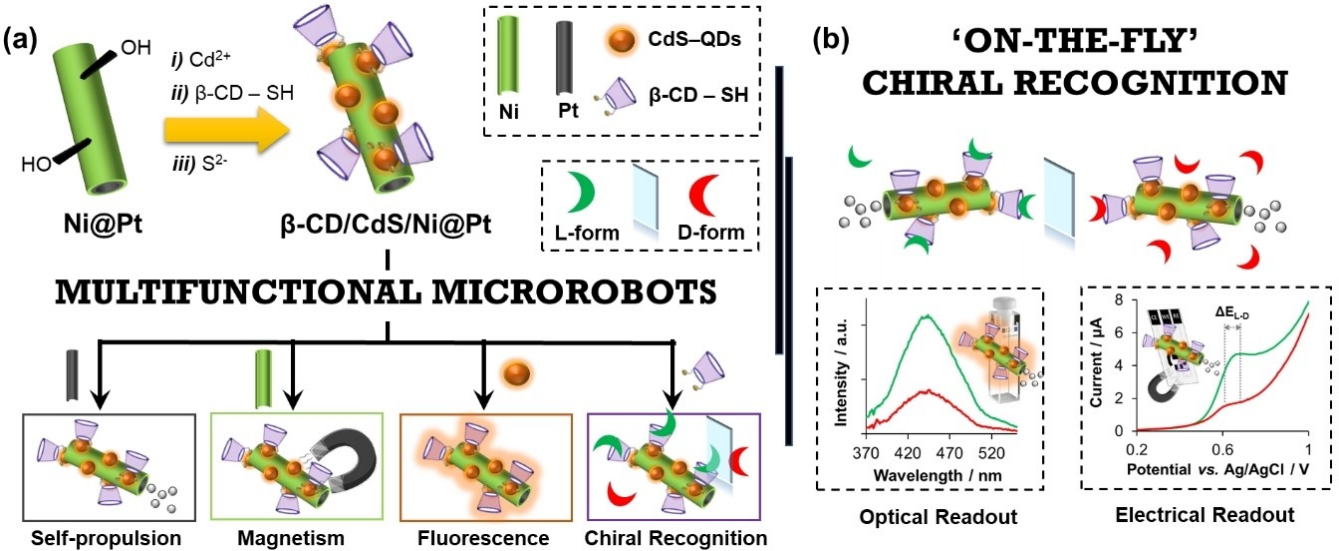
Self‐propelled chiral‐magneto‐fluorescent microrobots. a) Schematic illustration of the surface engineering carried out upon Ni@Pt microrockets to achieve the multifunctional β‐CD/CdS/Ni@Pt MRs. b) Bimodal (optical and electrical) “enantiorecognition‐on‐the‐fly” of chiral targets via supramolecular host–guest interactions.

Herein, a straightforward and generic synthetic procedure has been devised via surface engineering to produce for the first‐time multifunctional MRs that simultaneously exhibit foremostly chiral recognition, but also fluorescent properties and the ability to be magnetically navigable and propelled in the presence of a chemical fuel (i.e., H_2_O_2_). For this goal, nickel‐coated platinum (Ni@Pt) microrockets were tuned with fluorescence CdS‐QDs and a supramolecular host biomolecule as β‐CD via a “one‐pot” synthesis, resulting in β‐CD/CdS/Ni@Pt MRs (see Scheme 1a for illustration). Their potential towards “on‐the‐fly” chiral recognition approaches has been interrogated for the first time by using Trp enantiomers as proof chiral targets, where the output signals derived from supramolecular host–guest interactions have been readout through a dual—optical and electrical—method (Scheme 1b).

## Results and Discussion

### Synthesis and Characterization of Self‐Propelled Chiral‐Magneto‐Fluorescent MRs

The surface engineering employed to produce the multifunctional MRs is illustrated in Scheme 1a. In brief, nickel coated platinum (Ni@Pt) microrockets were firstly synthesized into the pores of a polycarbonate membrane using a conventional electrodeposition method^[9]^ (Figure S1). Then, they were tuned with fluorescent CdS‐QDs and a thiolated β‐CD ‐used to build‐in the chiral supramolecular recognition onto the moving MRs‐ via a “one‐pot” synthesis (see Supporting Information for more experimental details). CdS‐QDs were incorporated on the walls of the MRs by taking advantage of the ion‐exchange capabilities of the hydroxyl groups on the Ni surface after activation in alkaline solution.[^21^, ^22^, ^23^] Moreover, CdS‐QDs not only acted as fluorescence moieties but also as nanotemplates for the immobilization of the thiolated β‐CD via S−S bond formation,^[24]^ resulting in the multifunctional β‐CD/CdS/Ni@Pt MRs. Remarkably, while Ni is commonly utilized only as a coating for the development of superparamagnetic‐based MRs,[^9^, ^25^] here, Ni was also utilized as the main material for further functionalization.

**Figure 1:**
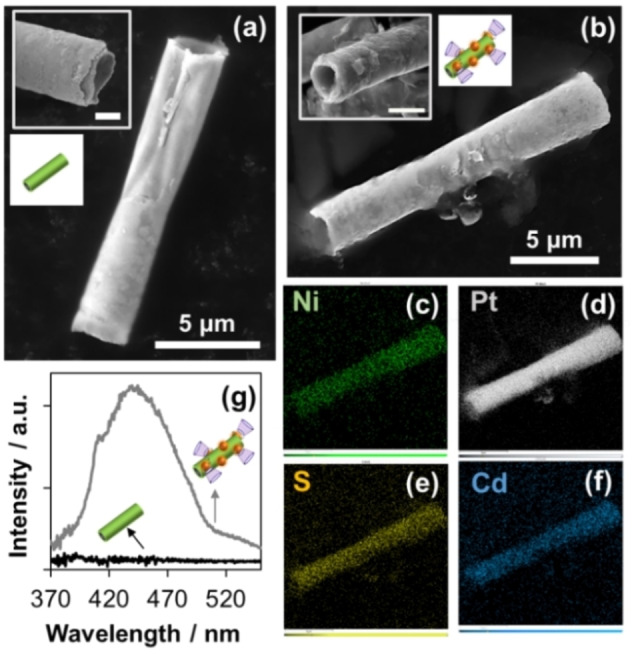
Material characterization of self‐propelled chiral‐magneto‐fluorescent microrobots. SEM images for a) pristine Ni@Pt microrockets and b) multifunctional β‐CD/CdS/Ni@Pt MRs (insets: SEM magnification, scale bar: 2 μm). EDX elemental mapping on β‐CD/CdS/Ni@Pt MRs for c) Ni, d) Pt, e) S and f) Cd. g) Emission spectra of pristine Ni@Pt microrockets (black line) and β‐CD/CdS/Ni@Pt MRs (grey line). Experimental conditions: *λ*_exc_=337 nm and *λ*_em_=440 nm.

The successful synthesis of the novel self‐propelled chiral‐magneto‐fluorescent MRs was confirmed by scanning electron microscopy (SEM), energy‐dispersive X‐ray spectroscopy (EDX) and fluorescent analyses (Figure 1) as well as ζ‐potential (Figure S2) and vibrating sample magnetometer (VSM) measurements (Figure S3). Figure 1a presents the side‐view SEM images of the pristine Ni@Pt microrockets showing a lateral size of 10–20 μm and a diameter of 2–3 μm, where homogeneous Ni outer and Pt inner layers were perfectly identified through top‐view (inset). Remarkably, a significant roughness increase was observed after functionalizing the Ni@Pt microrockets with CdS‐QDs and β‐CD (Figure 1b), while the tubular form remained unaltered for their subsequent proper motion (inset). The surface chemical composition of the multifunctional MRs was addressed by means of EDX, as shown in Figure 1c–f. The elemental mapping composition revealed the presence of Ni, Pt, Cd and S, which verifies the successful fabrication of the multifunctional β‐CD/CdS/Ni@Pt MRs. The intrinsic optical properties of CdS‐QDs were also explored to elucidate the fluorescence activity of the multifunctional MRs. Figure 1g displays the emission spectrum of β‐CD/CdS/Ni@Pt MRs, with a maximum intensity at 440 nm (excitation light, *λ*_ex_=337 nm). Importantly, no fluorescence activity was observed at this given wavelength when pristine Ni@Pt microrockets were utilized as a control, pointing out the successful transfer of the optical properties of CdS‐QDs on the multifunctional MRs.

The ζ‐potential of pristine and functionalized MRs in aqueous dispersions are shown in Figure S2. For comparison, functionalized MRs without the presence of β‐CD were also measured as a control experiment. A significant increase in this value from −8.52 to −6.37 (control) and +0.64 mV was reached after functionalizing the microrockets, which further evidenced the successful combination of CdS‐QDs and β‐CD with the Ni@Pt microrockets. As expected, close values between pristine and control MRs were obtained owing to the nature of the chemistry (ion‐exchange) involved for CdS‐QDs incorporation, since the functional groups of the Ni@Pt substrate remain unaltered.^[21]^ Further, Figure S3 depicts the VSM magnetic properties of the micromachines at room temperature before and after functionalization. Both curves appear S‐shaped over the applied magnetic field with magnetic remanences nearly to zero, indicating that there was almost no residual magnetization once the external magnetic field was removed. This demonstrates that the superparamagnetic properties of Ni@Pt microrockets remained unaltered after their functionalization. As a result, the rotation and translation of the multifunctional MRs in pure water was possible to be driven by using a transversal rotating magnetic field generated through a home‐made magnetic controller system (Figure S4 and Movie S1). Despite in this work superparamagnetic features were mainly included in the multifunctional MRs to enable their collection through an external magnet, this magnetic field‐controlled locomotion is very promising (e.g., for cargo transport, microsurgery, and electronic applications).[^26^, ^27^, ^28^]

**Figure 2:**
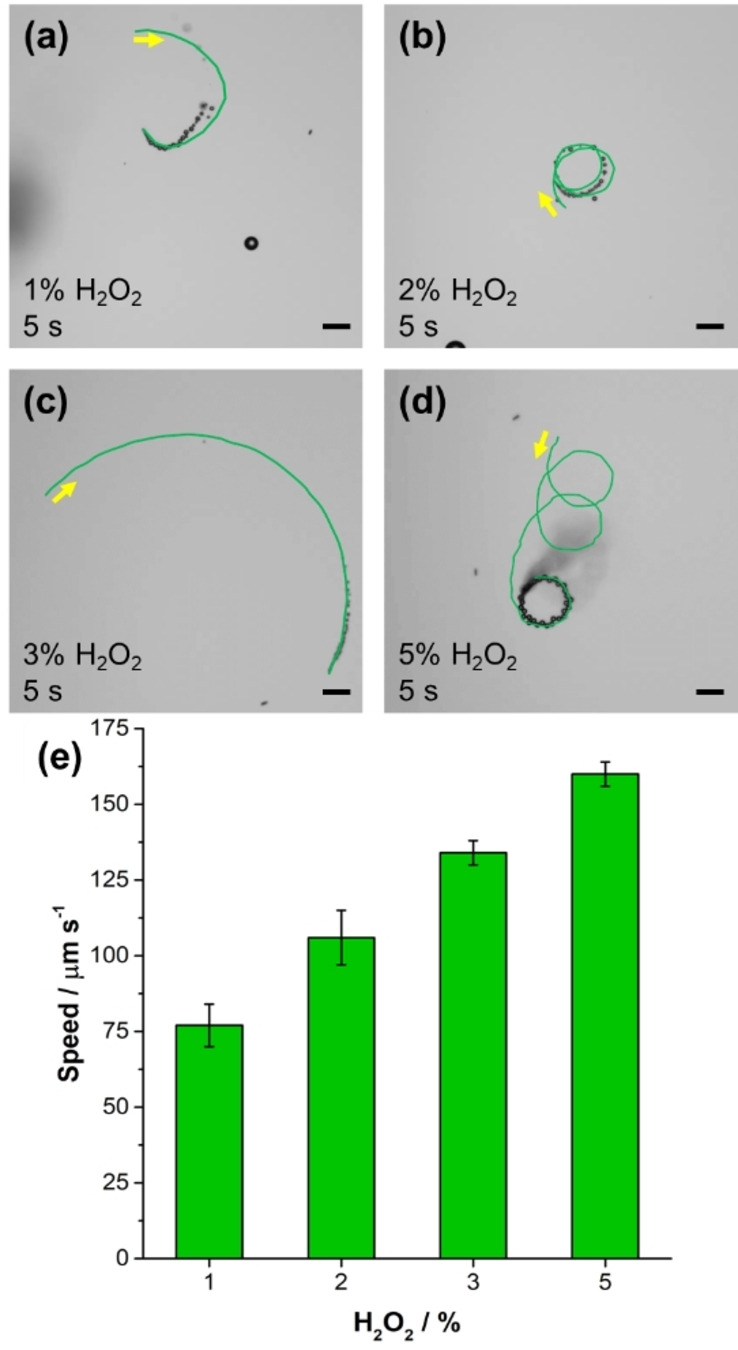
Motion behavior of self‐propelled chiral‐magneto‐fluorescent MRs. Trajectories recorded for 5 s in a) 1 %, b) 2 %, c) 3 % and d) 5 % H_2_O_2_. Yellow arrows indicate the movement direction of MRs. e) MRs speed as function of H_2_O_2_ concentration. Scale bars: 40 μm.

The rocket‐like design of the multifunctional MRs allows their strong bubble‐induced self‐propulsion through the decomposition of H_2_O_2_ fuel catalyzed by the Pt inner layer.^[29]^ Thus, the influence of fuel concentration on the velocity of the multifunctional MRs was explored using different H_2_O_2_ concentrations (from 1 % to 5 %). The bubble propulsion of the multifunctional MRs at increasing fuel concentration is clearly visualized in the micrographs of Figure 2a–d, where circular trajectories over 5 s recording in either a clockwise (a–c) or a counterclockwise (d) direction are shown (Movie S2). As expected, longer trajectories were obtained for higher fuel concentration as the speed of the multifunctional MRs significantly increased from ≈75 μm s^−1^ at 1 % H_2_O_2_ to ≈165 μm s^−1^ at 5 % H_2_O_2_ (Figure 2e). It is worth noting that a similar speed was measured for the Ni@Pt microrockets before and after the functionalization (138±28 vs. 134±4 μm s^−1^ using 3 % H_2_O_2_ for pristine and functionalized micromachines, respectively), justifying our choice to focus only on the motion behavior of the functionalized ones. Owing to the biotoxicity of H_2_O_2_ at high concentrations, 1 % H_2_O_2_ has been chosen for propelling the multifunctional MRs in the following “enantiorecognition‐on‐the‐fly” experiments.

### “On‐the‐Fly” Chiral Recognition of Trp Enantiomers with Bimodal Readout

Having verified the successful preparation of self‐propelled chiral‐magneto‐fluorescent MRs, their potential use towards “enantiorecognition‐on‐the‐fly” analyses was explored. As a first demonstration of applicability, Trp was used as a model chiral target by taking advantage of the different affinity of β‐CD to supramolecularly host amino acids enantiomers.^[20]^ In particular, Trp is one of many important chiral amino acids—the building blocks in protein biosynthesis—which play a predominant role in the physiological and pharmaceutical metabolisms in biological systems.^[19]^ The resulting enantiomeric output signals derived from the supramolecular host–guest interactions between β‐CD and L‐/D‐Trp were monitored through a dual fluorescence and electrochemical readout method.

**Figure 3:**
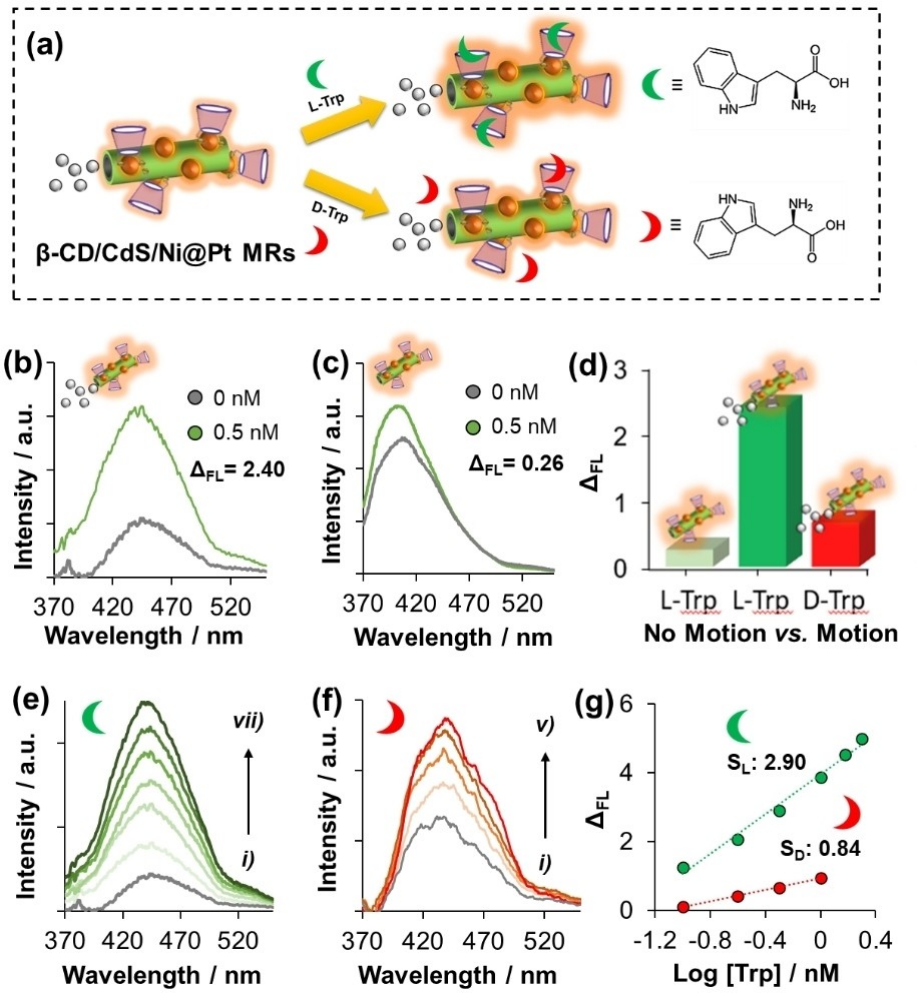
Self‐propelled chiral‐magneto‐fluorescent microrobots for the “enantiorecognitio‐on‐the‐fly” of Trp enantiomers with optical readout. a) Schematic illustration of the recognition of Trp enantiomers by the multifunctional MRs. Optical analyses of the multifunctional MRs before (grey line) and after adding 0.5 nM of L‐Trp (green line) in b) 1 % H_2_O_2_ fuel (motion) and c) pure H_2_O (no motion). d) Histogram showing the Δ_FL_ values achieved in pure H_2_O vs. 1 % H_2_O_2_ for 0.5 nM of L‐Trp and 0.5 nM of D‐Trp (from left to right, respectively). “On‐the‐fly” optical enantiosensing of e) L‐Trp and f) D‐Trp (from i) 0.1 to vii) 2.5 nM) with g) their corresponding calibration curves. *λ*_em_: 440 nm.

On one hand, the self‐propelled chiral‐magneto‐fluorescent MRs were used as chiral recognition selectors for the optical “on‐the‐fly” determination of Trp enantiomers (Figure 3a). The fluorometric assay was based on adding in a quartz cuvette a fixed amount of multifunctional MRs containing different concentrations of either L‐Trp or D‐Trp at the nM range. Subsequently, a drop of fuel was added to achieve a 1 % H_2_O_2_ solution, which was used to promote the supramolecular β‐CD/Trp complex formation. The fluorescence (FL) signals of the loaded β‐CD/CdS‐QDs/Ni@Pt MRs were measured after a 1 min incubation. Then, the intensity changes were represented by means of Δ_FL_=(*I*_Trp_−*I*_0_)/*I*_0_, where *I*_0_ and *I*_Trp_ are the fluorescence intensity obtained before and after adding a fixed concentration of Trp enantiomers, respectively.

As shown in Figure 3b, the intensity signal of the multifunctional MRs was highly increased after adding 0.5 nM of L‐Trp due to the supramolecular complex formation, yielding to a Δ_FL_ value of 2.40. Since Trp can act as an electron donor,^[30]^ a partial FL increase through the activation of the surface states of the pair β‐CD/CdS‐QDs via host–guest amino acid binding (i.e., Trp) is expected.^[20]^ This demonstrates the key role of β‐CD to host the biomolecular target in their cavity. Figure 3c shows a control experiment carried out in pure H_2_O (without adding fuel). Importantly, the Δ_FL_ value was reduced by 89 % (from 2.40 to 0.26) when the multifunctional MRs were not in movement (in other words, when they act as passive supramolecular carriers in pure H_2_O). These excellent results point out the added value of “on‐the‐fly” supramolecular recognition since self‐propulsion can notably accelerate this chemical operation. In addition, the enantioselectivity of the multifunctional MRs was compared by exposing them to 0.5 mM of either L‐Trp or D‐Trp. As depicted in Figure 3d, a Δ_FL_ value as low as 0.67 was reached using the D‐form of the amino acid, indicating an enantioselectivity of ≈3.5 times higher for L‐Trp. These results are in good agreement with the reported ratio of inclusion constants between β‐CD and Trp enantiomers (*K*_L‐Trp_ */K*_D‐Trp_), which has been determined to be around 3.3.^[31]^ The larger *K* value of L‐Trp is ascribed to the easier H‐bond formation between the −OH groups on the edge of β‐CD and the indole group of L‐Trp, when compared with D‐Trp.^[32]^ Thus, the higher FL intensities reached by L‐Trp also demonstrates the better binding affinity of the multifunctional MRs carrying the host β‐CD towards L‐Trp than D‐Trp.

Such unusually large difference in the FL response of the two enantiomers was also utilized to interrogate the potential of the self‐propelled chiral‐magneto‐fluorescent MRs as optical enantiobiosensing platforms. Figure 3e–f present the effect on the fluorescence spectrum of the multifunctional MRs with increasing concentrations of L‐Trp and D‐Trp (from 0.1 to 2.5 nM), respectively. Although both Trp enantiomers significantly enhanced the fluorescence signal, this enhancement was found to be highly enantioselective (71 %)—note the different photosensitivity (slope values) from the calibration curve of Figure 3g—for L‐Trp.

**Figure 4:**
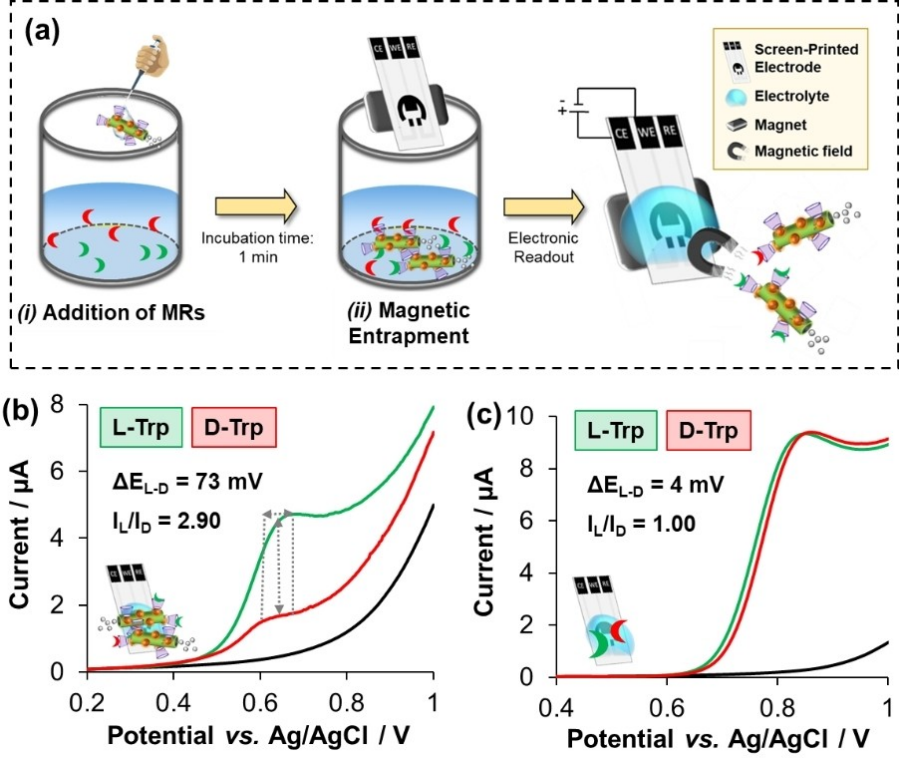
Self‐propelled chiral‐magneto‐fluorescent microrobots for the “enantiorecognition‐on‐the‐fly” of Trp enantiomers with electrical readout. a) Schematic illustration of the electroanalytical assay which relies on: i) adding a fixed amount of multifunctional MRs in a 1 % H_2_O_2_ aqueous solution containing 100 μM of Trp enantiomers and aged for 1 min to induce the supramolecular binding formation; ii) magnetically trapping the loaded MRs on a magneto carbon‐based screen‐printed electrode for b) their subsequently electrochemical enantiodiscrimination via lineal sweep voltammetry. c) Direct determination of L‐Trp and D‐Trp at the naked screen‐printed electrode (blank experiment). Experimental conditions: Electrolyte: PBS at pH 7.2; Scan rate: 50 mV s^−1^.

On the other hand, the electrochemical performance of the multifunctional MRs towards the discrimination of Trp enantiomers was also investigated in order to further prove the concept of “enantiorecognition‐on‐the‐fly”. The electroanalytical assay, which is summarized in Figure 4a, was based on incubating a fixed concentration of the multifunctional MRs in a 1 % H_2_O_2_ solution containing 0.1 μM of either L‐Trp or D‐Trp for 1 min. After that, the loaded MRs were magnetically trapped on a screen‐printed carbon electrode (SPCE) through a small neodymium magnet. Thus, the multifunctional MRs were utilized here as supramolecular cargo carriers for pre‐concentrating the chiral targets on the electrode surface by exploiting their magnetic properties. The electrochemical enantioselective readout relies on the electroactive properties of Trp enantiomers to be oxidized by applying an optimum redox potential input. As shown in the lineal sweep voltammograms from Figure 4b, a clear electrochemical discrimination between the Trp enantiomers by means of redox potential (*E*_p_) and peak current (*I*_p_) was reached. Recognition efficiencies of 73 mV and 2.90 were estimated by peak‐to‐peak separation (Δ*E*_L‐D_) and peak current ratio (*I*_L_/*I*_D_) means, respectively. The difference on these pivotal electrochemical parameters implies that the self‐propelled chiral‐magneto‐fluorescent MRs can interact with L‐Trp and D‐Trp in different ways, as it was expected from the fluorometric assay, demonstrating again that the multifunctional MRs preferentially recognizes L‐Trp. Owing to the hydrophobic nature of the host β‐CD cavity, the less polar part of the guest isomers (indole group) fits its cavity, while the more polar part (amino group) is exposed to the terminal −OH groups on the host rims. Thus, the chiral discrimination mechanism is associated to the more favorable affinity of the −OH groups from the host to form H‐bonds with the amino groups of L‐Trp (rather than D‐Trp).^[32]^ Importantly, a blank experiment with the naked SPCE exhibited overlapped voltammograms when the two Trp enantiomers were directly added in the electrolyte medium (Figure 4c), indicating that no stereoselective recognition occurred at the naked electrode (without the presence of multifunctional MRs). Finally, a control experiment was also run without the presence of fuel (MRs acting as passive supramolecular carriers in pure H_2_O). As shown in Figure S5, weak *I*_p_ signals with almost no chiral discrimination values (*I*_L_/*I*_D_ of 1.32 and Δ*E*_L‐D_ of 10 mV) resulted from this experiment, demonstrating again the added value of self‐propulsion to rapidly pre‐concentrate (incubation time: 1 min), and therefore discriminate, the chiral targets on the electrode surface.

Thereby, these results demonstrate the versatility of the multifunctional MRs which enable the possibility of discriminating Trp enantiomers via a dual (optical and electrical) method.

## Conclusion

In summary, an unprecedented self‐propelled chiral‐magneto‐fluorescent microrobot made of nickel‐coated platinum (Ni@Pt) microrockets, quantum dots (CdS‐QDs) and a host β‐cyclodextrin (β‐CD) has been devised via a simple eco‐friendly “one‐pot” synthesis to elucidate the yet undisclosed concept of “enantiorecognition‐on‐the‐fly”. The characterization of the resulting β‐CD/CdS/Ni@Pt MRs has demonstrated that i) the strong magnetization and navigability of the Ni core, ii) the self‐propelled motion allowed by Pt in presence of H_2_O_2_ fuel, iii) the fluorescent properties of CdS‐QDs, as well as iv) the supramolecular enantiorecognition capabilities of β‐CD biomolecules are retained in this multifunctional micromachine.

As a first demonstration of applicability, the multifunctional MRs have shown promising results in the field of analytical chemistry as new supramolecular cargo platforms for “on‐the‐fly” discrimination of chiral biomolecules (i.e., TRP enantiomers) using a dual (optical and electrochemical) readout method. It is expected that the combined chiral, magnetic and fluorescent functionalities in one self‐propelled micromachine would enable the engineering of unique smart host vehicles—which could be manipulated using an external magnet field—for new insights not only in the field of micro/nanomotors, but also in chiral (bio)sensors, (opto)electronic devices and biomedical applications by employing different readout methods.

## Conflict of interest

The authors declare no conflict of interest.

1

## Supporting information

As a service to our authors and readers, this journal provides supporting information supplied by the authors. Such materials are peer reviewed and may be re‐organized for online delivery, but are not copy‐edited or typeset. Technical support issues arising from supporting information (other than missing files) should be addressed to the authors.

Supporting Information

Supporting Information

Supporting Information

## Data Availability

The data that support the findings of this study are available from the corresponding author upon reasonable request.
