# Supplementary material for: Self‐Propelled Multifunctional Microrobots Harboring Chiral Supramolecular Selectors for “Enantiorecognition‐on‐the‐Fly”
Source: Angew Chem Int Ed Engl. 2022 Feb 9;61(14):e202116090. doi: 10.1002/anie.202116090 (PMC9304198; doi:10.1002/anie.202116090)
Supplement: Supplementary file 1 — Supporting Information [file ANIE-61-0-s003.pdf]

Angewandte Chemie
Eine Zeitschrift der Gesellschaft Deutscher Chemiker

Supporting Information

Self-Propelled Multifunctional Microrobots Harboring Chiral Supramolecular Selectors for "Enantiorecognition-on-the-Fly"

*J. Muñoz, M. Urso, M. Pumera**

Author Contributions

J.M. Conceptualization:Lead; Data curation:Equal; Formal analysis:Lead; Writing – original draft:Lead
M.U. Data curation:Supporting; Formal analysis:Supporting; Writing – review & editing:Supporting
M.P. Conceptualization:Lead; Resources:Lead; Supervision:Lead; Writing – review & editing:Lead

Supporting Information

Table of Contents

1. **Experimental Section**

1.1. *Chemicals & Materials*

1.2. *Synthesis of self-propelled chiral-magneto-fluorescent MRs*

1.3. *Apparatus & Procedures*

2. **Supporting Figures**

Figure S1. *Illustration of Ni@Pt MRs fabrication*

Figure S2. *ζ-potential of pristine and functionalized MRs*

Figure S3. *VSM of pristine and functionalized MRs*

Figure S4. *Magnetic field-controlled navigation of functionalized MRs*

Figure S5. *LSV employing passive MRs (control experiment)*

3. **References**

1. Experimental Section

1.1. Chemicals & Materials

$Cd(NO_3)_2 \cdot 4H_2O$ (Cd^{2+} precursor), NaHS (S^{2-} precursor), tryptophan enantiomers (L– and D–Trp), sodium dodecyl sulfate (SDS) and H_2O_2 were purchased from Sigma-Aldrich (St. Louis, MO, USA). Per-6-thio-β-cyclodextrin (thiolated β–CD) was acquired from CycloLab (Budapest, Hungary). All the aqueous solutions were prepared using deionized water. All the rest of reagents were of at least analytical grade and used as received.

1.2. Synthesis of self-propelled chiral-magneto-fluorescent MRs

Firstly, self-propelled Ni@Pt superparamagnetic microrockets were fabricated by a membrane-assisted electrodeposition method. A 90 nm thick Ag layer was deposited by electron-beam evaporation on a Whatman Cyclopore polycarbonate membrane (3 μm pore size) stuck on a piece of copper tape to fabricate a working electrode. Then, a three-electrode electrochemical cell was assembled using the prepared working electrode, a Pt counter electrode and an Ag/AgCl (1 M KCl) reference electrode. The cell was connected to a Metrohm AUTOLAB potentiostat. Commercial Ni and Pt plating solution (Singapore) were used. Ni outer layer deposition was carried out by chronoamperometry at a constant applied potential of -1.0 V *vs.* Ag/AgCl for 60 s. Pt inner layer deposition was performed by chronopotentiometry at a constant current of -20 mA for 500 s. The membrane was detached from the copper tape, carefully washed with deionized water and hand polished with an alumina slurry to remove the Ag layer. Subsequently, the membrane was sonicated for 5 min in deionized water to remove unwanted microparticulate and dissolved in dichloromethane under vigorous agitation to release the Ni@Pt microrockets, as schematically illustrated in **Figure S1**. These were cleaned with different solvents at increasing polarity (dichloromethane, isopropanol, ethanol) and with deionized water via vortex agitation for 5 min followed by centrifugation at 5000 rpm for

3 min to separate the solvent from microrockets. Microrockets were finally stored in 1 ml deionized water solution at room temperature.

Prior to the functionalization with with fluorescent CdS–QDs and chiral β-CD selector, Ni@Pt microrocets were activated with a 1.0 M KOH aqueous solution in order to promote the formation of hydroxyl groups on Ni surface.[1] Afterwards, the activated Ni@Pt microrockets were functionalized with CdS–QDs and β-CD *via* a 'one-pot' synthesis as follows: *i)* Ni@Pt microrockets were suspended in a 1 mL KOH aqueous solution (pH 11) containing 2.5 mM $Cd(NO_3)_2{\cdot}4H_2O$ (the Cd^{2+} precursor) and *ii)* 1.0 mM of per-6-thio-β-cyclodextrin (thiolated β-CD) and sonicated for 30 min. In this step, the driving force relies on the electrostatic interactions between the hydroxyl groups present on the Ni surface and the Cd^{2+} metal ion precursor.[2] Then, *iii)* the mixture was precipitated by adding 1 mL of a 1.25 mM solution of $Na_2S{\cdot}9H_2O$ (S^{2-} precursor). The molar ratio of Cd^{2+}/S^{2-} was fixed at 2:1 as was previously reported.[3] The solution was aged overnight at room temperature under soft agitation, inducing the *in situ* nucleation of CdS–QDs in the proximity of the Ni@Pt microrockets. The strength of the CdS-thiol interactions provides the basis for obtaining robustly-structured self-propelled MRs that simultaneously possess magnetic, fluorescent, and chiral properties in combination with self-propulsion.[4] The obtained β-CD/CdS/Ni@Pt MRs were removed magnetically, washed several times with Milli-Q water and ethanol and then re-dispersed in water to obtain the self-propelled chiral-magneto-fluorescent MRs stock suspension, which was stored at room temperature.

1.3. Apparatus & Procedures

The morphological and chemical composition (elemental mapping) analyses of the MRs were carried out by using a SEM (TESCAN MIRA 3 XMU) coupled to an EDX detector (Oxford Instruments). The magnetic behavior was measured *via* VSM system (Quantum

Design VersaLab) at room temperature with a maximum applied magnetic field of 5 kOe. Optical measurements were acquired on a Jasco FP-8300 spectrofluorometer. The emission spectra of the multifunctional MRs in the presence of Trp enantiomers were recorded at 440 nm by excitation at 337 nm. Self-propulsion of MRs was recorded using a Nikon ECLIPSE Ti2 inverted optical microscope equipped with a Hamamatsu digital camera C13440-20CU. In a typical experiment, an aqueous suspension of MRs, 0.1 w% SDS and H_2O_2 at different concentrations (1, 2, 3, 5%) was dropped on a glass slide. NIS Elements Advanced Research software was used to record the bubble propulsion of MRs at 15 fps. Videos were manually analyzed using Fiji software to get microrockets trajectories and measure their speed. Magnetic field-controlled navigation experiments were conducted through a Nikon Ts2R inverted microscope equipped with a Basler acA1920-155uc camera using a home-made magnetic controller system with three orthogonal coil pairs in a 3D-printed polylactic acid (PLA) backbone generating a rotating magnetic field. Navigation experiments were performed with an applied magnetic field of 3 mT at different frequencies (10, 20, 30, 40, and 50 Hz). Videos were recorded by Pylon Viewer software at 15 fps, and automatically tracked by NIS Elements Advanced Research software. Electrochemical experiments were made by means of LSV in an AutoLab Metrohm PGSTAT 204 potentiostat/galvanostat equipped with NOVA 2.1 software. Electrochemical experiments were carried out using disposable screen-printed carbon electrodes (SPCEs, Zensor), including carbon pseudoreference, counter, and working electrodes. A neodymium magnet was used to promote the attraction of the multifunctional MRs to the SPCEs. A phosphate buffered solution (PBS, pH 7.2) was employed for the measurements.

In order to avoid the effect of the fuel, optical measurements were carried out by substracting the background employing the solvent (water containing 1% in H_2O_2 or pure

water for the blank), while the electrochemical assay was performed outside the electrochemical cell for supramolecular interaction after being trapped by the electrode with the help of an external magnetic field.

2. Supporting Figures

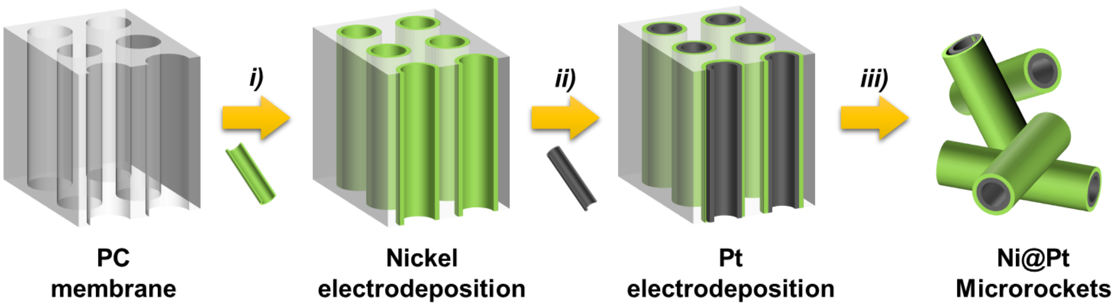

Figure S1. Schematic illustration of Ni@Pt microrockets fabrication. *i)* Ni (bias of -1.0 V (*vs.* Ag/AgCl) for 60 s) and *ii)* Pt (current of -20 mA for 500 s) layers were electrodeposited into the pores of a polycarbonate (PC) membrane and then *iii)* released from the PC membrane using dichloromethane.

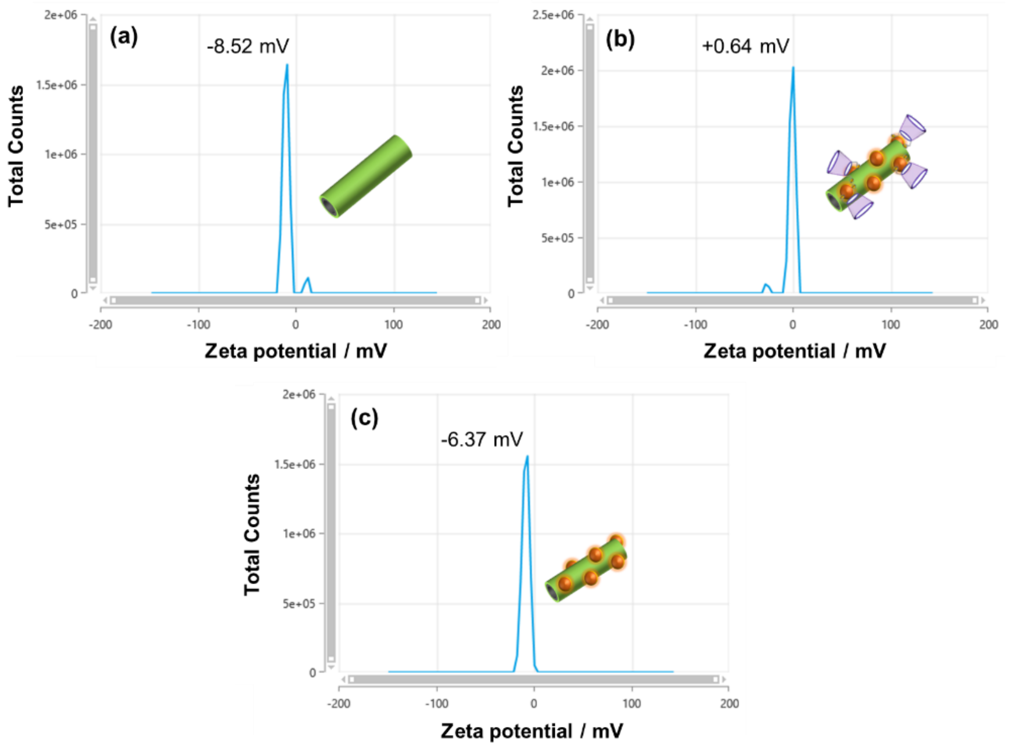

Figure S2. Density charge characteristics of micromachines. ζ-potential measurements of **(a)** pristine Ni@Pt microrockets and **(b)** β-CD/CdS/Ni@Pt MRs. **(c)** Control experiment employing MRs without the presence of the thiolated β-CD derivative (CdS/Ni@Pt MRs).

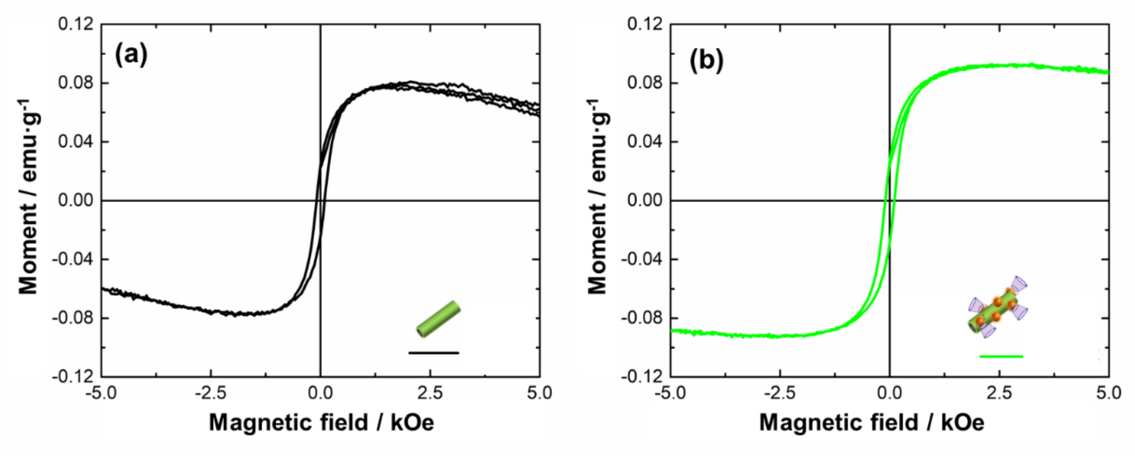

Figure S3. Superparamagnetic characteristics of micromachines. VSM characterization of **(a)** pristine Ni@Pt microrockets and **(b)** β-CD/CdS/Ni@Pt MRs.

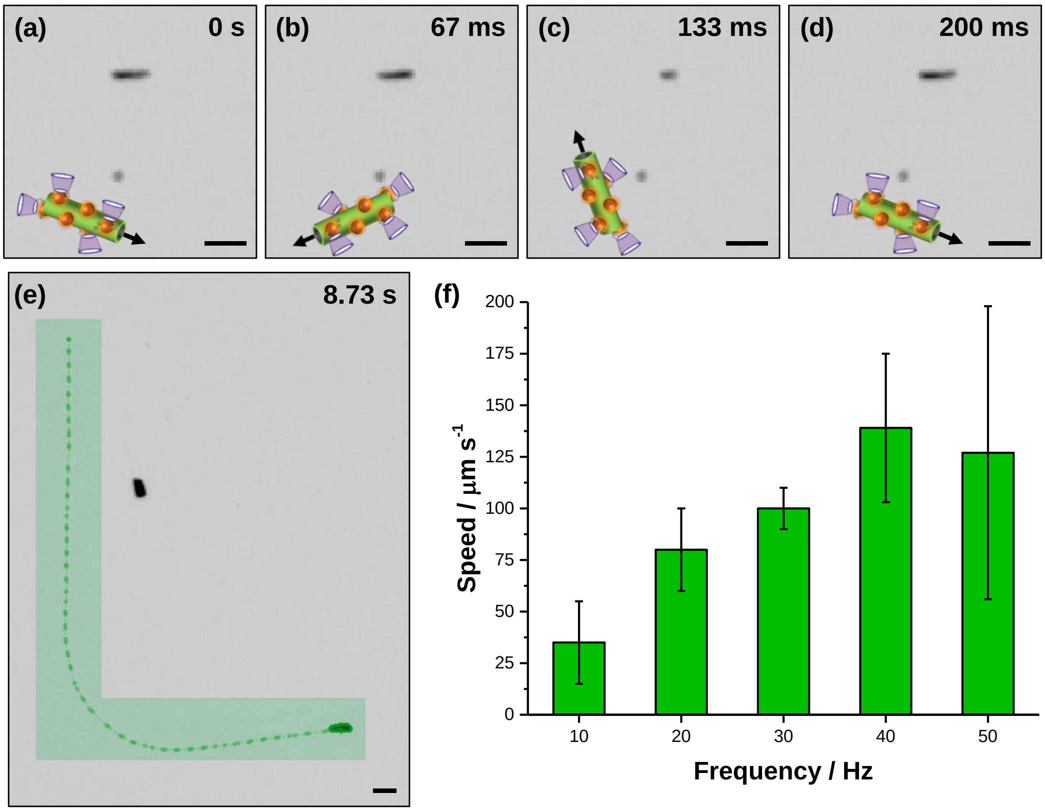

Figure S4. Magnetic field-controlled navigation of self-propelled chiral-magneto-fluorescence MRs. **(a-d)** Time-lapse images of a MR on the xy plane showing its rotation on the yz plane, **(e)** whose trajectory was finely controlled spelling the letter "L" on the xy plane as an example. **(f)** Navigation speed as function of applied magnetic field frequency. According to this, the maximum frequency allowed to perform magnetic experiments is 40 Hz and, beyond that, most of the MRs are not able to rotate at all. Scale bars: 15 µm.

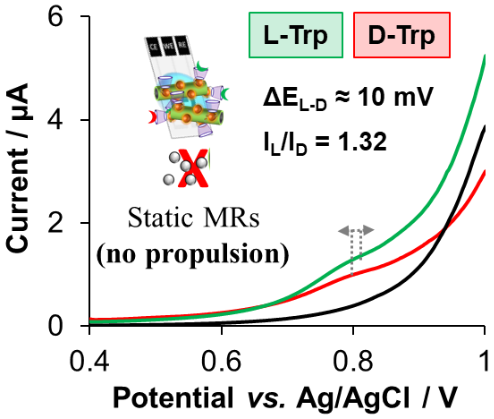

Figure S5. LSV resulting from the control experiment employing passive MRs (without motion) towards the electrochemical discrimination of Trp enantiomers. Experimental conditions: Electrolyte: PBS at pH 7.2; Scan rate: 50 mV·s^{-1}; Concentration of Trp enantiomers: 100 nM; Incubation time: 1 min.

References

[1] S. L. Medway, C. A. Lucas, A. Kowal, R. J. Nichols, D. Johnson, *J. Electroanal. Chem.* **2006**, *587*, 172–181.

[2] J. Muñoz, J. Bastos-Arrieta, M. Muñoz, D. Muraviev, F. Céspedes, M. Baeza, *J. Mater. Sci.* **2016**, *51*, 1610–1619.

[3] M. Li, H. Zhou, H. Zhang, P. Sun, K. Yi, M. Wang, Z. Dong, S. Xu, *J. Lumin.* **2010**, *130*, 1935–1940.

[4] P. Thangadurai, S. Balaji, P. T. Manoharan, *Nanotechnology* **2008**, *19*, 435708.
